# Stakeholder development of an implementation strategy for fall prevention in Norwegian home care – a qualitative co-creation approach

**DOI:** 10.1186/s12913-023-10394-x

**Published:** 2023-12-11

**Authors:** Siv Linnerud, Linda Aimee Hartford Kvael, Birgitte Graverholt, Gro Idland, Kristin Taraldsen, Therese Brovold

**Affiliations:** 1 Department of Rehabilitation Science and Health Technology, Faculty of Health Sciences, OsloMet - Oslo Metropolitan University, P.O. Box 4, St. Olavs plass, Oslo, N-0130 Norway; 2 Department of Housing and Ageing Research, Norwegian Social Science, OsloMet - Oslo Metropolitan University, P.O. Box 4, St. Olavs plass, Oslo, N-0130 Norway; 3 Department of Health and Functioning, Western Norway University of Applied Sciences, P.O. Box 7030, N-5020 Bergen, Norway; 4 Agency of Health, The municipality of Oslo, N-0130 Oslo, Norway

**Keywords:** Implementation, Implementation strategy, Co-creation, Fall prevention, Stakeholder engagement, Uptake of evidence, Older adults, Home care services

## Abstract

**Background:**

The uptake of fall prevention evidence has been slow and limited in home care services. Involving stakeholders in the implementation process is suggested as a method to successfully tailor implementation strategies. The aim of this study was to develop an implementation strategy for fall prevention, targeting healthcare providers working in home care services.

**Methods:**

This study used an explorative qualitative approach in a five-step co-creation process to involve researchers, service users, and healthcare providers. The first two steps consisted of workshops. This was followed by focus group interviews and individual interviews with key informants as steps three and four. Data from the first four steps were analyzed using reflexive thematic analysis. The fifth and final step was a workshop finalizing a strategy for implementing fall prevention evidence in home health services.

**Results:**

Overall, our findings, resulted in an implementation strategy for fall prevention with four components: (1) Empower leaders to facilitate implementation, operationalized through what managers pay attention to regularly, resource priorities, and time spent on fall prevention, (2) Establish implementation teams, consisting of multidisciplinary healthcare providers from different levels of the organization, with formalized responsibility for implementation, (3) Tailor dual competence improvement, reflecting the need for knowledge and skills for fall prevention and implementation among healthcare providers and users, and (4) Provide implementation support, representing guidance through the implementation process.

**Conclusions:**

This study advances our understanding of implementation in home care services. Implementation of fall prevention requires an implementation strategy involving a blend of essential components targeting leaders, competent healthcare providers and users, and establishing structures enhancing the implementation process.

**Supplementary Information:**

The online version contains supplementary material available at 10.1186/s12913-023-10394-x.

## Background

Over the last decade, implementation of research evidence into clinical practice has been slow [1], including the implementation of research evidence for fall prevention among older adults [2–4]. Based on decades with research on effective ways to prevent falls, researchers agree on the importance of identifying and assessing risk factors and manage effective intervention such as strength- and balance training to prevent fall [5]. Despite this, one-third of adults aged 65 years and above still experience falls annually, causing injuries and an increased risk of morbidity. Falls among older adults are also considered costly for society as it often increases the need for health care services [6].

Preventing falls is highly relevant for community-based home care services, as they provide medical services and care to older adults living in their own homes [7]. Still, the implementation of fall prevention evidence is considered a complex process that require systematic work to identify barriers and facilitators at the patient-, provider-, organization-, and policy levels of health services [8, 9]. Research highlights lack of time, resources and local motivation as well-known barriers to implementation in community-based healthcare. Similarly, the involvement of employees and scenario-based training are considered facilitators that promote implementation processes [10]. Implementation represents a set of methods or techniques used to enhance the adoption, implementation and sustainability of a clinical intervention [11] and represent the ‘how to’ component of a clinical intervention.

A generally recognized principle in implementation is to tailor the strategy to fit the local setting, in terms of specific barriers and facilitators [12]. For instance, active learning techniques, which increase the activity and involvement of employees, such as tailored teaching and workshops, are commonly used implementation strategies to enhance uptake of research evidence [10, 13].

One of the most commonly used frameworks to address local key barriers and facilitators is the Consolidation Framework for Implementation Research (CFIR) [14]. CFIR was developed based on existing theories for implementation and comprises five constructs to represent a foundation for understanding implementation: *Intervention characteristics*, e.g. the complexity of fall prevention interventions, *Inner setting* e.g. culture for prevention of falls in the services, *Outer setting*, e.g. external, worldwide or national recommendations for fall prevention, *Process* e.g. how the implementation is conducted, *and Characteristics of individuals*, e.g. healthcare providers knowledge and beliefs about fall prevention [14]. CFIR is often applied in combination with the implementation strategies of Powell. A tool matches suitable implementation strategies to the barriers identified through CFIR [15].

To identify local barriers and facilitators for implementation, and tailor implementation strategies, involvement of stakeholders has been highlighted as vital [16]. Involving both service users and other stakeholders such as health care providers in tailoring the implementation strategy could increase the possibility of succeeding with the implementation [1]. However, in implementation science there is no consensus on how involvement of relevant stakeholders can best happen [17]. Co-creation is one method that contributes to active involving [18], described as a collaborative process to generate knowledge by involving researchers, service users, and other stakeholders [19]. The process is considered a non-hierarchical process, whereby stakeholders bring valuable insights and expertise into the collaboration. Given the challenge of research evidence finding its way into clinical practice, co-creation could be a suitable method for involving stakeholders to develop, tailor, or adapt different implementation strategies and increase the potential of succeeding in delivering evidence based care [19].

The aim of this study was to develop an implementation strategy for fall prevention in Norwegian home care services.

## Method

### Design

This study was designed as a multi-method qualitative co-creation process, with a mix of workshops, focus group interviews and individual interviews. Participants were researchers, health providers and service users.

### Context

This study occurred in the home care services in two city districts of Oslo municipality, Norway. Both city districts had experience in implementing fall prevention interventions including a multifactorial risk assessment form in home care services. Home care services provide home nursing, rehabilitation, and practical assistance to inhabitants living in their own homes, delivered by multidisciplinary healthcare providers. The homecare services are provided and primarily financed by municipalities and the services constitute the lowest level of formal care in the Norwegian healthcare system. All citizens are entitled to these services, regardless of age, gender, socioeconomic status or other characteristics [20]. Citizens who receive home care services pay a small fee for some of the services, such as for practical assistance, while traditional nursing care is free of charge. The Health and Care Services Act (2011) regulates the services provided by the municipalities and these include health care, health promotion and disease and injury prevention, including preventing falls among the inhabitants.

This study is part of the research project FALLPREVENT - Implementation of evidence-based fall prevention programs in health care services in Norway, as a preparation to a cluster randomized trial.

### Characteristics of participants

The sample consisted of thirty participants (20% male), strategically selected through purposeful sampling [21], selected for their previous experience with fall prevention. We included two researchers with backgrounds in fall prevention and implementation science, two older adults who had experienced a fall and were users of home care services, one professional user representative from “the pensioners association”, and twenty-five healthcare providers from different levels of home care services (eleven physiotherapists, three occupational therapists, one medical doctor, eight nurses and two assistant nurses). Users were recruited by health care providers working in the two city-districts and healthcare providers were recruited by their employer. Researchers, the professional user representative, and the medical doctor were recruited through the authors’ network.

### The co-creation process

**Fig. 1:**
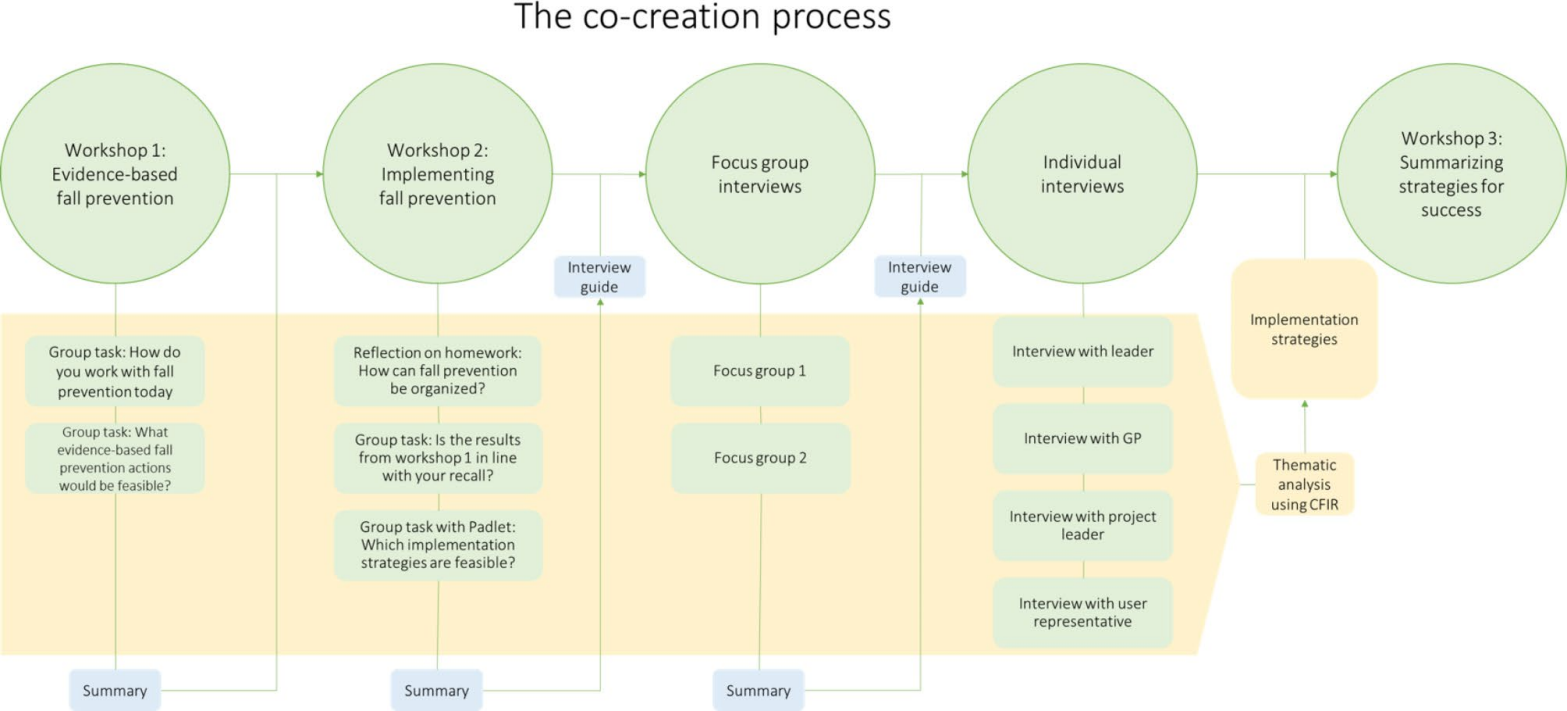
The steps of the co-creation process

We organized the co-creation process as a five-step process with three workshops (steps 1, 2, and 5), two focus group interviews (step 3), and four individual interviews (step 4) (Fig. 1) inspired by Engell and colleagues [22]. Using multiple data collection methods provided a broader insight and let us elaborate findings with other stakeholders. The first four steps were conducted during the spring of 2020, and step 5 was conducted in October 2022. All steps were digitally organized and recorded using Zoom due to Covid-19 restrictions. The workshops had a duration between 2 and 3.5 h, while focus groups and individual interviews lasted 1 h. After each step of the process, the authors wrote a summary, which gave direction and content for the next step. The summaries were validated by the participants at the beginning of the next step of the process.

The first two steps included healthcare providers, users, and researchers. During the workshops the participants were presented with research evidence for preventing falls, how fall prevention evidence could be translated into practice and introduced to implementation strategies. Participants were divided into two small groups with healthcare professionals and service users from their own city districts and one researcher in each group. The groups discussed current fall-preventive practices, what evidence already implemented into practice and the feasibility of different evidence and possible implementation strategies. After the first step, participants were asked to reflect on how fall prevention could be organized in home care services, as preparation for Step 2.

In the third step, the summary from the first two workshops was explored in two focus group interviews with healthcare providers. After the first three steps, the need for broader perspective into the following three topics was still needed: leadership in the implementation process, the GP’s role in fall prevention, and understanding of user experiences. Thus, as a fourth step we conducted individual interviews with key informants: (1) one manager in home care services, (2) a medical doctor working as a GP, (3) a physiotherapist with long experience in project management of a fall prevention project, and (4) a professional user representative with experience as a faller and with fall prevention through previous work in the health services. Topic guide for focus group interviews is presented as additionally file 2 and interview guide for individual interviews as additional file 3.

**Fig. 2:**
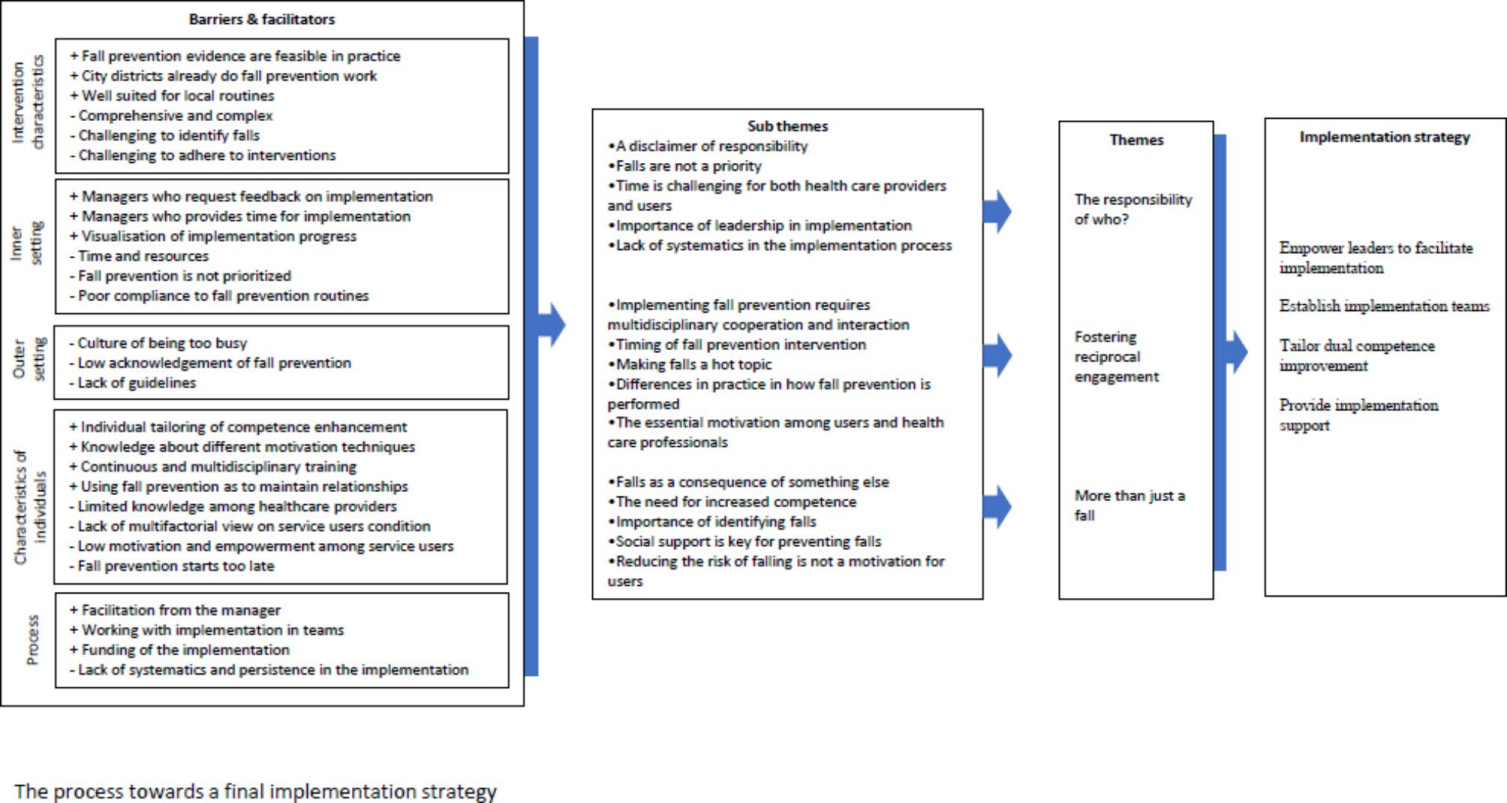
Logic model of barriers, facilitators, themes and implementation strategies

Before the fifth step, all material from step one to four was transcribed verbatim by the first author and uploaded into NVivo software for qualitative analysis. In accordance with reflexive thematic analysis as described by Braun and Clarke, three of the authors (SL, TB, and LAHK) independently read the material to become familiar with the material [23]. The material was systematically examined for barriers and facilitators and coded within the framework of CFIR. The barriers and facilitators were emerged into meaningful units, before searching for themes across the material. The process of analysing was iterative ultimately resulting in three main themes and revealing the three overarching barriers. The overarching barriers were matched with implementation strategies, supported by the facilitators and a tentative implementation strategy emerged (Fig. 2).

In the final workshop (step 5), the participants were divided into random groups and asked to discuss and adjust the implementation strategy through three group tasks. A final draft of the implementation strategy was generated by the authors and sent to all participants by e-mail for final comments and amendments before the strategy was finalised. Se supplementary file 1 for full description of content.

## Results

### An implementation strategy with four components

The co-creation process resulted in an implementation strategy with four components: (1) Empower leaders to facilitate implementation, (2) Establish implementation teams, (3) Tailor dual competence improvement, and (4) Provide implementation support. The components are not mutually exclusive, and all four components should be taken into consideration when implementing fall prevention evidence in home care services.

### Empower leaders to facilitate implementation

Empower leaders to facilitate implementation implies managers who show dedication to the implementation by investing time in pursuing the organization’s needs and influencing employees’ behaviours and attitudes.

The participants highlighted that commitment among managers were vital for successful implementation and organizational change. All highlighted the importance of leader involvement and engagement to be able to maintain a focus on fall prevention in a hectic workday. The participants preferred managers who engaged in fall prevention on daily basis, asked questions, and who kept fall prevention consistently on the agenda. Managers who requested updates on progress about fall prevention, helped the participants justify their time spent on tasks to prevent falls. For example, one of the key informants from the individual interviews stated:

“*Having managers that ask what is going on, says a lot about the culture of the workplace, right? Is this [fall prevention] something I should prioritize in my work, or rather not”.* (Participant ID 22, individual interview)

In addition, managers who acknowledge staff who prioritized fall prevention and show adherence to evidence set the standards for fall prevention and validated the managers commitment. One of the nurses in the second workshop stated:

*Being recognised, taken seriously, listened to and asked to demonstrate your competencies. I think that’s important and serves as a motivator.* (Participant ID 6, workshop)

The participants described workdays with a variety of essential tasks related to the health and care of the older adults, where fall prevention was described as time-consuming. The participants expressed a need for more time to prioritize and implement fall prevention interventions. Prioritizing the use of time and resources on preventing falls and having leaders put fall prevention on the agenda was essential for showing commitment. For example, one of the physiotherapists in the focus group expressed:

*“… it`s really important that it [fall prevention] is firmly embedded among the managers, that’s the blessing I need to spend worktime on implementation”.* (Participant ID 16, focus group)

The need for dedicated time for implementation was also highlighted as a part of the resource required in the organization. Dedicated time for continuous implementation should be integrated with other essential activities, as change is an ongoing activity. One nurse highlighted this in the third workshop by stating:

*“There is always something that needs to be implemented. There is always something new”.* (Participant ID 24, workshop)

This continuous focus on implementation was also expressed as a barrier, where the participants expressed the demands of continuous change as exhausting, with a risk of implementation fatigue. The participants also described the critical role of their managers commitment to establish systematic implementation of preventing falls. The lack of systematicity was pointed out as a barrier to implementing fall prevention. Having managers who was committed to the work and actively participating in the implementation process was described as a factor for success. For example, one of the physiotherapists participating in the second workshop stated:

*“And you need managers who shows willingness to change and who creates structure that facilitate the actions”.* (Participant ID 8, workshop)

Having committed managers influence employees into prioritizing the prevention of falls and helped adhere to recommendations. Committed leadership is expressed through what managers and leaders pay attention to on a regular basis, priorities of resource and time spent on fall prevention.

### Establishing implementation teams

Implementation teams define a group of stakeholders with multidisciplinary background, who are formally responsible to supervise, manage and support implementation processes.

During the co-creation process, it became clear that implementing fall prevention required a defined group of staff having implementation of fall prevention as their main task, with dedicated time, and a systematic multidisciplinary approach. Involving staff from various disciplines who have different perspectives on fall prevention, was crucial for the implementation. One of the nurses participating in the first workshops stated:

*“When we work multidisciplinary, we play each other well by sharing competencies and keep it [implementation] in the spotlight, that’s what that makes interventions last”.* (Participant ID 6, workshop)

In one of the city-districts, they had established an implementation team, working together in different levels in the organization. One physiotherapist from a focus group interview said:

*“We have a separate team dedicated to fall prevention. The team consists of five managers, two physiotherapists, a practice development nurse, and a health service supervisor. They all work with different areas of fall prevention and strategies for implementation in home care services”.* (Participant ID 16, focus group)

The team was viewed as a true resource to implement fall prevention measures, by actively helping out with challenges and contributing to progress. The same physiotherapist as above further expressed:

*“What is so good about the dedicated team is that it sort of opens up for working with implementation, if it holds the right persons”.* (Participant ID 16, focus group)

Other informants conveyed similar experiences. They realised how vital it was to establish functional fall prevention teams in each city district. Implementation teams were also recommended as a method to transfer and retain implementation competence within the services in case of turnover.

Having a systematic approach to implementation, in addition to implementation competence, seemed to be lacking in the city districts. One of the key informants, stated the need for someone to be responsible and accountable for implementation. In contrast, the managers presented a model where the responsibility for implementation was often given to a single employee working with direct care to users and where the leader would have limited oversights of implementation. The participants also stated how important it was that the implementation teams knew its responsibility and what it required from each participant in an implementation team.

Accordingly, establishing implementation teams with dedicated time and consisting of multidisciplinary health providers could be a sustainable way for the implementation of fall prevention.

### Tailor dual competence improvement

Competence improvement involves increasing the knowledge, attitudes, and skills in both fall prevention and implementation. All participants agreed on those two pillars being necessary for successful implementation of fall prevention evidence.

This requirement for developing higher competence among staff was emphasised as a necessary addition to a highly qualified and dedicated group of providers. Specifically, the participants highly commended their colleagues for the work they did but emphasised how critical it was that structures within the organisation helped them keep a sharp focus on fall prevention and implementation. One of the nurses stated:

*“They are our colleagues, and they are highly skilled, all with bachelor’s degrees. This is about keeping and supporting the awareness and attitudes. "* (Participant ID 6, workshop).

Despite the acknowledgement of their colleagues, the participants agreed that campaigning for fall prevention and implementation requires actions on different levels, to adjust capacity building almost to individual level. One of the nurses stated:

*“Our colleagues hold varying levels of competence, so there must be room for individualized training. People have different mindsets towards fall prevention which manifests in how they solve the different fall-prevention measures such as screening, documenting and so on. So yes, it’s important that there is room for individualized training, I think”.* (Participant ID 11, focus group)

Several methods were suggested to increase the competence among healthcare providers and users. One suggestion was to hold presentations about fall prevention at senior centres or other meeting places for healthcare providers and users. Another method suggested was to use next-of-kin to promote motivation for preventing falls. However, the essential method highlighted by the participants was working one-on-one with each user or guiding co-workers.

One of the challenges related to preventing falls among older adults was to identify falls. Health providers experienced a lack of reported falls that did not cause injury. The GP reported that people rarely reported falls unless prompted:

*“It is very rare that they [patients] tell me about their falls. Fallers most often don’t make an appointment for this reason. Rather, this is reported to me by the home care services or I get to know this through discharge notes from the ER or hospital. A few times, I’ve heard about a fall from a daughter or son”.* (Participant ID 19, individual interview)

More information on falls, having knowledge about falls not being a natural part of the aging process, how to report falls, when and to whom, was highlighted as essential information for users. Having healthcare providers asking about falls, repeatedly, was also described as a way to get older adults to talk about falls. One of the key informants, the professional user representative, stated her reflection on reporting of falls:

*“… they [users]may have had two or three falls before telling us. So, there is something about talking about falls and create awareness of how important it is to inform us, even when if it went well”.* (Participant ID 22, individual interview)

Other participants also highlighted motivation as an important strategy to succeed with implementation. One physiotherapist stated:

*“I think staff need to see the gains of all the work, such as screening, and that it actually leads to actions”.* (Participant ID 10, focus group)

Through the material, the participants agreed on the importance of preventing falls, but few found fall prevention to be their primary task except for the physiotherapists. Experiencing benefits of preventing falls was recommended to understand the importance and help motivate those involved in fall prevention work. This was also highlighted as a motivation for users, to actually experience the advantages of fall prevention and for the information to be provided at an opportune time from a user perspective. Providing information before a fall or before the users developed high risk of falling was highlighted, rather than after the fall. The GP stated:

*“It’s not easy to motivate for [lifestyle] change in older adults so, maybe the chance of preventing falls is higher if we start talking about it before they reach a certain age”.* (Participant ID 19, individual interview)

Presenting relevant information about falls to older adults at an early stage, before falling, was suggested as an inspiration and motivation for users to change their actions to prevent falls. The participants also made suggestions on the content of information to prevent falls. For example, some did not know the effect of exercise to prevent falls, what exercises needed to be performed and how often. Participants involved were from city districts where alcohol was a significant contributor to fall risk and so focusing on relevant risk factors was highlighted as important.

Healthcare providers and users indicated the need for enhances competence on how to effectively implement fall prevention.

### Provide implementation support

Based on previous experience and research, the authors recommended a fourth component, *support of the implementation*, to provide the city districts with necessary help through the process that could be transferred and used in other settings and when implementing other research evidence. This component represents guidance and supervision to the services on the process of implementation.

Support during implementation became visible also through the co-creation process, especially the need for knowledge about how to plan and conduct implementation. All participants found implementation challenging. Often, the implementation method of trial and error was used. One of the leaders said:

*“For implementation of new practices, we work by the trial-and-error method, because no one knows how it’s supposed to be”.* (Participant ID 21, individual interview)

Implementation did not occur as a prioritized activity, and implementation experiences from one field was typically not transferred to other fields. One of the key informants, the project leader, described the importance of having someone coordinating the implementation and reminding and requiring progress. She said:

“*The coordinating role that I hold, is very… I think it’s essential. […] Because fall prevention is a quite demanding task”.* (Participant ID 20, individual interviews)

Support of the implementation would also be a strategy, targeting the lack of systematicity that was described as a barrier related to committed leadership and the needed implementation competence within the services. The support should be provided to implementation teams, to improve their implementation competence. To guide the implementation process, we suggest using well known models or frameworks for implementation, such as the Knowledge-to-Action framework [24].

## Discussion

To our knowledge, this is the first study to co-create an implementation strategy designed to increase uptake of fall prevention evidence in home care services. The implementation strategy was developed through combining the insight and expertise of healthcare providers, users and researcher. The overall results indicate the importance of empower leaders to facilitate implementation, establishing implementation teams, the need for tailor dual competence among healthcare providers and users, and provide implementation support.

Our findings underline the value of having committed leaders who facilitate the implementation process, expressed through what they pay attention to and request feedback on. The participants mainly addressed the role of their closest leaders, mangers who had the daily responsibility of their tasks, while the role of top leaders were rarely addressed. The engagement of leaders is within the CFIR framework, an indicator of organizational readiness for implementation, and includes the leader’s commitment, involvement, and accountability within the implementation [14]. How managers engage and are involved in the implementation process can influence the staff to prioritize the implementation. Managers responsibility for employees, operations, and budgets are essential as they distribute important resources, such as extra time spent on implementation, and can control the implementation by adjusting these factors [25]. Specifically, managers hold a key role in establishing implementation teams, prioritizing what tasks the teams spend time on, as well as competence improvement among the entire staff. Schein and Schein (2016) define what managers and leaders pay attention to as the “value of leaders”, and one of six mechanisms influencing the organizational climate and perceptions of employees [26]. What managers and leaders pay attention to is demonstrated through the content of their communication with the staff. Our results confirms that specially managers can directly impact priorities the staff make during a hectic clinical workday, but the key role of the top leaders’ role and strategic leadership was not addressed by the participants. The strategic leadership plays an important part in setting the course in local plans for further development of the services. The included city districts had years of experience implementing fall prevention, and as this had been on the agenda for a long time, they might have underestimated the role of the top leaders.

In line with previous research [27, 28] our results yield a need for more collaboration within the implementation efforts, where implementation teams are recognized as a successful initiative. Making the implementation a collaborative effort by involving different healthcare providers, was suggested as a key to success. Metz and Bartley (2020) support the need to rely on multiple actors due to the complexity of implementation [29]. Furthermore, our results also underline the success of the group leading the collaborative initiative is dependent on involving the right team members. The team members must be chosen thoughtfully as they are to be considered role models and influencers for the implementation [14, 29].

Implementation teams have been considered a structure for supporting the implementation and a strategy for increasing stakeholder engagement [29]. Setting up teams that comprises multidisciplinary members from different levels of the organization was indicated by the participants in our study as a way to embrace the breadth of fall prevention and increase commitment. The multidisciplinary team could be a way to increase commitment and engagement to prevent falls, and to even underline that the responsibility of fall prevention sits across the multidisciplinary team. This is supported by Metz and Bartley, as they introduce members who represent different perspectives [29]. To formalise the responsibility of the implementation teams was also suggested by the participants, which is supported by Metz and Bartley. Acknowledging members of the implementation team as implementation leaders is considered essential in the CFIR framework.

We found the need for increased competence among users and healthcare providers in both fall prevention and implementation. The preferred method for increasing competence in fall prevention was for health care providers to work one-to-one with users and one-on-one with supervising colleagues. Implementing fall prevention evidence includes the individuals’ attitudes, knowledge, and beliefs about fall prevention, and is influenced by the access to fall prevention evidence [14]. Additionally, to prevent falls it requires skills to transform evidence into actions, which requires belief in one’s own capability to execute fall prevention. In line with CFIR, Powell presents several implementation strategies targeting competence improvement, such as educational meetings, developing educational material, and making training dynamic [12]. Gransjøen (2022) presented a variety of methods to increase competence, such as teaching, meetings, workshops, and gatherings, to successfully implement guidelines and recommendations in municipal healthcare [10]. Use of similar methods was also supported by the systematic review of the Norwegian Knowledge Centre for the Health Services, where it found educational meetings increased the adherence to clinical practice guidelines with moderate certainty [30].

Schultes and colleagues called for a comprehensive set of knowledge and skills for practical implementation [31]. Our participants shared this view, as they mostly used the method of trial and error and felt uncertainty in not having any formal competencies in systematic implementation. Although previous experiences with implementation projects is a valuable asset, it needs to be supported by formal and systematic implementation of competencies [31].

To increase implementation competence over time, providing implementation support was suggested. Providing implementation support to the services could enhance their systematic in implementation projects. Within implementation science, models such as the Knowledge to Action Model and Quality Implementation Framework describe or guide the process of translating research into practice [32]. Despite thorough descriptions of steps, in these frameworks, their application to real-world implementation projects is resource demanding. Using implementation advisors to support the process is another implementation strategy highlighted by the ERIC compilation [12]. The implementation process is also included in the CFIR framework, but the 2009 CFIR domains provide no action steps for the process [14]. This was recently reviewed in the updated version, including key activities such as assessing needs to the Process domain of the framework. The Process domain does not describe how the implementation process should occur but is more of a reminder of what should occur during implementation [33].

An important part of implementation in general is the context where the implementation takes place. Contextual factors could influence the success of the implementation strategy, important, as what appear to be a barrier in one context could occur as a facilitator in another and contextual factors could influence the success of implementation [34]. In CFIR, the context represents the constructs inner- and outer setting, representing both local conditions, attitudes, communication and culture amongst others [14]. How to empower leaders to facilitate implementation, who to participate in the implementation teams and how to tailor dual competence improvement should be tailored to local context. One way to achieve this is by using models of framework supporting the systematic in the implementation process as recommended for support through the implementation process [24].

### Strengths and limitations

There are several strengths and limitations to the current study. The findings represent the view of 30 participants, including researchers, users and healthcare providers from two city districts of Oslo municipality. Oslo is a municipality with 15 city districts and the largest city in Norway, but the work to prevent falls among community-dwelling older adults is often quite similar despite the size and organizations of services within municipalities. The city district included in this study had experience in implementing fall prevention and their experiences have informed the implementation strategy. Therefore, we believe the results might be transferrable to other municipalities, limited to homecare services.

Although we included stakeholders representing both healthcare providers, researchers and service users, the distribution of stakeholders were mostly healthcare providers including a larger share of physiotherapists. To get a more nuanced perspective, four key informants, (one manager in home care services, a medical doctor, a project leader, and a professional user representative), were included. We tried to include more service users to the sample but faced some challenges with sickness among some of them. To ensure that the voice of service users was heard, there were specific tasks during the workshops targeting service users’ experiences. Our sample included both males and females, but mostly female participants, which mirrors the gender distribution in Norwegian healthcare.

We could have applied additional methods to increase the insight into implementation of fall prevention evidence. However, our multiple sources of data and key informants likely supports the credibility and trustworthiness of our results. Further, to increase trustworthiness, we undertook member checking from each step, where summaries were provided to and checked by participants. To promote reflexivity, the first author wrote a researcher memo. The authors’ preconceptions were that evidence-based fall prevention evidence has not been sufficiently implemented in the municipal health services. The authors of this paper represent nurses (SL and BG) and physiotherapists (TB, LAHK, KT and GI) with broad clinical and/or research experience within municipal healthcare, fall prevention, quality improvement and implementation science. Combining these perspectives and experiences, we believe has been an advantage in keeping an analytic distance to the data material.

## Conclusions

This study advances our understanding of implementation of fall prevention in home care services. Our findings suggest an implementation strategy consisting of empowering leaders to facilitate implementation, establishing implementation teams, tailoring dual competence improvement, and providing implementation support as important factors when implementing fall prevention evidence in home care services. Empowering leaders to facilitate implementation highlights the importance of what managers pay attention to, priority of resource and time used on fall prevention. The implementation should be led by teams consisting of multidisciplinary healthcare providers from different levels of the organization who have the formal responsibility for providing commitment and progress in the process. Tailoring competence improvement should target both fall prevention and implementation knowledge and should be provided for both healthcare providers and users. Lastly, providing implementation support through supervision and guidance is needed to ensure a systematic implementation process. With a growing interest in how to succeed with uptake of fall prevention evidence, we believe our results represent a possible blend of essential components to consider for implementation processes in home care services.

### Electronic supplementary material

Below is the link to the electronic supplementary material.

Supplementary Material 1

Supplementary Material 2

Supplementary Material 3

Supplementary Material 4

## Data Availability

The dataset supporting the conclusions of this study can be obtain by contacting the first author, Siv Linnerud.

## List of abbreviations

CFIR: Consolidation Framework for Implementation Research
ERIC: Expert Recommendations for Implementing Change
