# Supplementary material for: Stakeholder development of an implementation strategy for fall prevention in Norwegian home care – a qualitative co-creation approach
Source: BMC Health Serv Res. 2023 Dec 11;23:1390. doi: 10.1186/s12913-023-10394-x (PMC10714538; doi:10.1186/s12913-023-10394-x)
Supplement: Supplementary file 1 — Supplementary Material 1 [file 12913_2023_10394_MOESM1_ESM.docx]

# Description of co-creation process

Description of the content in the co-creation process in FallPrevent, including details on group tasks during workshops and topic addressed in interviews. The process was conducted digitally, using Zoom, and recording the entire process.

| **Steps** | **Participants and duration** | **Content** |
| --- | --- | --- |
| Step 1:  Workshop 1 | Participants:   - Healthcare providers - Users - Researchers   Duration: 3.5 hours | **Welcome and a technical introduction to Zoom**  The facilitator welcomed all participants to the workshop and gave a brief introduction to Zoom: how to mute/unmute, turn on/off camera and raise hands for questions.  **Presentation of all participants and getting to know each-other**  A presentation round was led by the facilitator, where participants gave a brief presentation of themselves and previous experience with fall prevention.  **An introduction to the project**  The facilitator presented the rational for the project, including aims and upcoming activities.  **Establishing principles for co-creation**  The facilitator gave an overview of the co-creation process and principles for the collaboration:   - All participants are equal parts - Respect for each other and other opinions - All contributions are just as important - Everyone is entitled to share their opinions   **Small group discussion: How is fall prevention carried out today?**  Participants were divided into two groups consisting of researchers and users and healthcare providers from the same city districts. The task was used to get the participants to reflect about todays practice and how falls are prevented today. Through the task, the groups were asked to discuss what was working, challenges in the work and experiences from users. One participant was chosen as the group leader, and each group had a co-facilitator present in the breakout room for questions and help during the task. In the end, the groups summarized their task in plenary.  **Presentation of fall prevention evidence**  One of the co-facilitators presented the evidence on fall prevention.  **Small group discussion: What is feasible in the city district?**  The participants were divided into the same groups as for the previous group task, and discussed the fall prevention evidence, what is feasible, what is not feasible and what changes need to be done to make it feasible in practice. Like the previous task, one participant was chosen as the group leader, and each group had a co-facilitator present in the breakout room for questions and help during the task. In the end, the groups summarized their answer to the task in plenary.  **Summary of the workshop**  At the end of the workshop a summary was provided in plenary by the facilitator. And as a homework before the second workshop, the participants were asked to reflect upon how fall prevention can be organized. |
| Between steps 1 and 2 |  | **Summary for validation**  After the workshop, the facilitator and co-facilitators wrote a summary of the workshop and group discussion, using recordings from the workshop. |
| Step 2:  Workshop 2 | Participants:   - Healthcare providers - Users - Researchers   Duration: 3.5 hours | **Welcome and presentation of all participants**  The facilitator started welcoming all participants to the second workshop and a quick introduction of all participants before the facilitator presented the agenda. The facilitator repeated the rationale for the project, the project aims and activities.  **Reflection on how to organize fall prevention**  A plenary reflection on the refection task from the homework, how fall prevention should be organized, were led by the facilitator and co-facilitators.  **Summary from workshop 1**  The facilitator presented the summary from workshop 1 and the participants validated summary and discussed the results in small group discussions.  **Small group discussion: Does the summary reflect your memory of workshop 1, and what is most critical to prioritize in fall prevention?**  Participants were divided into similar groups as in the first workshop, discussing the group task. One participant was chosen as the group leader, and each group had a co-facilitator present in the breakout room for questions and help during the task. In the end, the groups summarized their task in plenary.  **Introduction to implementation**  The facilitator provided an introduction to implementation to the participants, presenting the evidence for implementation strategies and methods to succeed with implementation. Participants were able to ask questions during the presentation.  **Small group discussion: What strategies are feasible to increase uptake of fall prevention evidence?**  The groups then discussed methods for increasing the uptake of fall prevention evidence, using tablet as a tool to facilitate the discussion. In tablet, different questions were provided to enhance the discussion. One participant was chosen to submit their discussion in Tablet, and through the task, each group had a co-facilitator present in the breakout room for questions and help during the task. In the end, the groups summarized their task in plenary.  **Summary**  At the end of the workshop a summary was provided in plenary by the facilitator. |
| Between steps 2 and 3 |  | **Summary for validation**  After the workshop, the facilitator and co-facilitators wrote a summary of the workshops using recordings from the workshop. The summary provided direction and content of step 3. |
| Step 3:  Focus group interviews | Participants:   - Healthcare providers   Duration: 1 hour | **Topics addressed in the focus groups:**   - Fall preventive work - How do you work to prevent falls in your everyday work? - How often do you do this? - How do you identify potential fallers? - Who do you collaborate with? - Any challenges related to this work? - Change of working method - If you learn about new guidelines/research within fall prevention, what makes you change the way you work? - How do you share this with colleagues? - What flexibility do you have to decide for yourself how to work? - Competence among employees - How can we increase competence among employees? - An example from the workshops was training, one-to-one or in a group, how would you like the training to be laid out? - What is feasible for your work situation? - Another example from the work seminars was that it was important to create excitement about fall prevention. What does it take for you to be motivated or excited about fall prevention? - Motivation of the user - How do you work to motivate users? - What are good means of increasing user motivation? - An example that came up in the workshops was the importance of the relationship with users and the opportunity to tailor interventions - what do you think about that? |
| Between steps 3 and 4 |  | **Summary for validation**  After the focus group interviews, the facilitator and co-facilitators summarized the focus group interviews, providing direction and content of step 4. |
| Step 4:  Individual interviews with key informants | Participants:  4 Key informants:   - one leader in home care services, - a medical doctor working as a GP - a physiotherapist with long experience in project management - a professional user representative   Duration: 1 hour for each individual interview | Topics addressed in interviews with all key informants:   - Own role in fall prevention - Collaboration with others to prevent falls - Motivation among users   The leader was addressed with questions related to:   - Fall prevention leadership - Leader commitment in practice - Facilitating implementation of fall prevention   GP were addressed with questions related to:   - Identifying falls - GPs role in future   The project manager was addressed with questions related to:   - Experiences with implementation - Successful strategies used in implementation previously - Facilitators for implementation   The professional user representative was addressed with questions related to:   - How to identify falls among users - Communication to prevent falls - Competence among users and healthcare providers   In the end all key informants were asked to describe the ideal method to implement fall prevention, if resources were no limitation. |
| Between steps 4 and 5 |  | **Analysis**  All the material from the previous steps were transcribed verbatim by the facilitator and uploaded to NVivo Software. Analysis was conducted in accordance with thematic analysis by Braun and Clarke (2022). In the first step, the facilitator and the co-facilitators became familiar with the material through reading it independently. Then, the material was systematically reviewed using the CFIR framework to code barriers and facilitators into the different constructs of CFIR. The codes were controlled for their fit to the constructs and emerged into more thorough categories – or meaningful units. In the third phase, we searched for themes across the material. The process of analysis was dynamic, by going back and forth in defining and identifying themes, resulting in three main themes revealing the three overarching barriers. The overarching barriers were matched with implementation strategies and a tentative implementation strategy emerged. |
| Step 5:  Workshop 3 | Participants:   - Healthcare providers - Users - Researchers   Duration: 2 hours | **Welcome, presentation and an introduction to the project.**  The facilitator started welcoming all participants to the third workshop, followed by an introduction of all participants. An introduction to the prog before the facilitator presented the agenda. The facilitator repeated the rationale for the project, aims and activities. Then a summary of the previous workshops was provided to the participant, giving the participants ability to validate the results.  **Plenary task: What is leader commitment operationalized in practice?**  Participants discussed in plenary leader commitment and the current results from the thematic analysis.  **Small group discussion:**  The participants were divided into two groups, each group working on one of the cases using Tablet. One participant was chosen to submit their discussion in Tablet, and through the task, each group had a co-facilitator present in the breakout room for questions and help during the task.  **Case 1**: In September the Worlds falls guidelines were published, recommending asking three questions to older adults to identify falls. You are implementing the use of these three questions among healthcare providers in home health services. How would you increase their competence to succeed with this?   - What do they need training in? - Who should provide the training? - How should the training be organized? - When and how often do they need training?   **Case 2**: In September this year, the Worlds Falls Guidelines were published, and you are responsible for implementing the guidelines in the city district. To succeed with the implementation, you are recommended to establishing an implementation team.   - What should be their tasks? - Who should participate in the team? - How often should they meet? - What support does the team need?   **Summary**  In the end, the groups summarized their case in plenary and the other group contributed with their input. The facilitator provided a summary for the workshop and how the results would be used in the implementation strategy. All participants consented to receiving an e-mail of the final draft for comments and adjustments before the strategy was finalized. |
| After step 5 |  | After step 5, the facilitator and co-facilitator generated a final draft of the implementation strategy. The draft was sent to the participants aby e-mail for comments and amendments. The facilitator and co-facilitators then finalized the strategy. |
