# Supplementary material for: Stakeholder development of an implementation strategy for fall prevention in Norwegian home care – a qualitative co-creation approach
Source: BMC Health Serv Res. 2023 Dec 11;23:1390. doi: 10.1186/s12913-023-10394-x (PMC10714538; doi:10.1186/s12913-023-10394-x)
Supplement: Supplementary file 2 — Supplementary Material 2 [file 12913_2023_10394_MOESM2_ESM.docx]

**Topic guide focus group interviews**

Welcome from the moderator:

The aim of this focus group is to discuss how we can implement fall prevention recommendation among community dwelling older adults. Previously, we have arranged two workshops where some of your colleagues have participated. In this focus group interview, we would like you to discuss the results from these workshops. We will provide you with some questions for discussion, and at times we will ask for more information or ask for your opinion if we have not heard from you. The session will be recorded, so please remember not to share sensitive information about persons not present.

Do you have any questions before we begin?

**Fall preventive work**

- Can you talk about how you work to prevent falls in your everyday work?
- How often do you do this?
- How do you identify potential fallers?
- Who do you collaborate with?
- Do you find any challenges related to this work?

**Change of working method**

- If you learn about new guidelines/research within fall prevention, what makes you change the way you work?
- How do you share this with your colleagues?
- What flexibility do you have to decide for yourself how to work?

**Competence among employees**

- What are your thoughts on how we can increase competence among employees?
- An example from the workshops was training, one-to-one or in a group, how would you prefer to receive training?
- What is feasible for your work situation?
- Another example from the workshops was that it is important to create excitement about fall prevention. What does it take for you to be motivated or excited about fall prevention?

**Motivation of service users**

- How do you work to motivate users?
- An example that came up in the workshops was the importance of the relationship with users and the opportunity to tailor interventions - what are your thoughts on this?
