# Supplementary material for: Stakeholder development of an implementation strategy for fall prevention in Norwegian home care – a qualitative co-creation approach
Source: BMC Health Serv Res. 2023 Dec 11;23:1390. doi: 10.1186/s12913-023-10394-x (PMC10714538; doi:10.1186/s12913-023-10394-x)
Supplement: Supplementary file 3 — Supplementary Material 3 [file 12913_2023_10394_MOESM3_ESM.docx]

**Interview guide individual interviews**

Welcome from the interviewer:

The aim of this interview is to explore how we can implement fall prevention recommendation among community dwelling older adults. You have already red the information paper and signed an informed consent for participation. Just as a repetition, it is voluntary to participate, and you can withdraw you consent at any time. The session will be recorded, so please remember not to share sensitive information about persons not present.

Do you have any questions before we begin?

Can you please present yourself with name, age and background?

**Questions for the leader**

- Can you please describe your role as a leader and how you work with fall prevention in your everyday work?
- Can you please share your thoughts on leader engagement in fall prevention?
- Can you describe how you facilitate for collaboration in fall preventive work?
- Can you please describe how you work with competence enhancement of fall prevention?
- If resources were not an obstacle, how would you organize the fall preventive work?

**Questions for the medical doctor**

- Can you please talk about how you work to prevent falls among older adults?
- Other healthcare providers often highlight the important role of doctors in fall prevention work, can you please share your thoughts on this?
- Can you describe how you collaborate with other healthcare providers to prevent falls?
- What are your experiences on how to succeed with motivating older adults to prevent falls?
- If resources were not an obstacle, how would you organize the fall preventive work?

**Questions for the project manager**

- Can you please talk about the fall prevention project you are leading?
- Can you please talk about how you have worked with implementation of the project in the city districts?
- Can you please share what have been a success in the project?
- Can you please share your experiences with motivating staff in fall preventive work?
- If resources were not an obstacle, how would you organize the fall preventive work?

**Questions for the professional user representative**

- Can you please talk about your experiences with falls and fall prevention?
- One challenge highlighted by healthcare providers, are motivating users. Can you please share your thoughts on how to motivate for fall prevention activities?
- Can you please share your thoughts on what fall prevention competencies are needed?
- If you would have experienced a fall, how do you want the home care services to take care of you?
- What is the ideal fall preventive offer/service?

All interviews were ended with the interview summarizing the main topics of the interview and checking if this was perceived correct. To allow for additional information, the interview ended the interview with asking: Is there anything else you would like to share?
